# Transcriptional and hormonal profiling uncovers the interactions between plant developmental stages and RNA virus infection

**DOI:** 10.1099/jgv.0.002023

**Published:** 2024-09-18

**Authors:** Izan Melero, Aurelio Gómez-Cadenas, Rubén González, Santiago F. Elena

**Affiliations:** 1 Instituto de Biología Integrativa de Sistemas (CSIC – Universitat de València), Paterna, 46182 València, Spain; 2 Departamento de Biología, Bioquímica y Ciencias Naturales, Universitat Jaume I, 12071 Castelló, Spain; 3 The Santa Fe Institute, Santa Fe, NM 87501, USA

**Keywords:** hormonal responses, host developmental stages, plant–virus interaction, reproductive fitness, response to infection, transcriptomics

## Abstract

*Arabidopsis thaliana* is more susceptible to certain viruses during its later developmental stages. The differential responses and the mechanisms behind this development-dependent susceptibility to infection are still not fully understood. Here we explored the outcome of a viral infection at different host developmental stages by studying the response of *A. thaliana* to infection with turnip mosaic virus at three developmental stages: juvenile vegetative, bolting, and mature flowering plants. We found that infected plants at later stages downregulate cell wall biosynthetic genes and that this downregulation may be one factor facilitating viral spread and systemic infection. We also found that, despite being more susceptible to infection, infected mature flowering plants were more fertile (i.e. produce more viable seeds) than juvenile vegetative and bolting infected plants; that is, plants infected at the reproductive stage have greater fitness than plants infected at earlier developmental stages. Moreover, treatment of mature plants with salicylic acid increased resistance to infection at the cost of significantly reducing fertility. Together, these observations support a negative trade-off between viral susceptibility and plant fertility. Our findings point towards a development-dependent tolerance to infection.

## Introduction

Host developmental stage influences susceptibility to pathogen infection in many natural systems [1,3]. The host developmental stage is one key factor causing host physiological and phenotypic heterogeneity, thus having a strong impact on the outcome of host–pathogen interactions and the epidemiological dynamics resulting from them [4]. Plant systems possess tightly regulated developmental transitions driven by both genetic and environmental cues [5,7]. The developmental stage of a plant influences its responses to both abiotic and biotic stresses [8]. However, the extent and nature of the developmental stage-dependent responses to pathogens varies across different pathosystems [9,10]. Plants commonly show an age-related resistance (ARR) response to biotic stresses, in which older plants display an increased resistance to pathogens compared to younger ones [11,13]. However, for numerous RNA viruses, mature plants are more susceptible to infection than their younger counterparts [14,15]. As obligate intracellular pathogens, viruses rely on their host’s resources to complete their replication cycle. To successfully replicate, viruses follow several evolutionary strategies to secure their own reproduction and survival at the cost of their hosts’ fitness, aiming to reach optimal levels of virulence and transmission [16]. To achieve this goal, viruses manipulate their hosts in different ways, from inducing host mortality or sterility to symptomless tolerance [17,18]. Interestingly, host developmental stages have been argued to alter these virulence–transmission relationships [2, 17,19].

In this work we studied the pathosystem comprising *Arabidopsis thaliana* Heynh. and turnip mosaic virus (TuMV; species *Potyvirus rapae*, genus *Potyvirus*, family *Potyviridae*). *A. thaliana* is an annual flowering plant, with a life cycle that shifts between a phase of vegetative growth and a phase of reproductive growth. Arabidopsis plants undergo a highly regulated flowering transition where resources must be reallocated from biomass accumulation to the formation of reproductive structures. Arabidopsis responses to external factors can differ depending on the developmental time during which plants encounter these situations [8,12, 17, 19]. This developmental influence is also observed in the host responses against viruses [14,15]. Plants’ defence responses comprise different mechanisms: from gene silencing to the expression of defence genes, or the synthesis of small molecules known as phytohormones [20]. TuMV belongs to the genus *Potyvirus* [21], which includes many viruses that cause significant losses in crops and agriculture. TuMV can infect over 300 plant species, most belonging to the family *Brassicaceae* [22] (which *A. thaliana* belongs to). *A. thaliana* is a self-fertilizing plant that can produce tens of thousands of seeds per plant [23]. Nevertheless, upon infection with TuMV, seed production is severely affected and can even be completely abolished [24,25]. Thus, TuMV can be considered a sterilizing pathogen, preventing the plant from producing offspring.

Here we sought to better understand how host developmental stages influence the hosts’ responses to viral infection. To do so, we studied the infection of TuMV in *A. thaliana* of the accession Col-0 at three different developmental stages: (i) the vegetative juvenile stage, where plants allocate resources to increase their size and mass (prebolting); (ii) bolting, an indicator of developmental transition from vegetative to reproductive investment [26]; and (iii) flowering, where mature plants reallocate resources to reproduction (postbolting). A total of 18 viral lineages were studied, derived either from a naïve TuMV isolate, TuMV-AS [27], or from an *A. thaliana*-preadapted one after 12 passages of experimental evolution in prebolting plants, TuMV-DV [28] (for further details concerning the experimental evolution, see [15]). Three viral lineages were established for each TuMV isolate at each of the three host developmental stages considered.

In order to achieve our goal, we used a combined transcriptomic and metabolomic (hormonal profiling) approach. We studied the differentially expressed genes (DEGs) upon infection with the different evolved TuMV lineages at the three aforementioned developmental stages, and the hormonal profiles of such interactions. Analysis of these results led us to hypothesize that salicylic acid (SA) accumulation and virus movement played crucial roles in the differences observed. Additionally, we studied the production of progeny of plants infected at these three developmental stages compared to mock-inoculated control plants. Our findings explored the different responses to viral infection and the impact of host developmental stage on the outcome of Arabidopsis–TuMV interactions, which point towards a development-dependent tolerance to virus infection.

## Methods

### Plant material and growth conditions

*A. thaliana* Col-0 were grown in a climatic chamber under a photoperiod of 16 h light (PAR of 125 µmol m^−2^ s^−1^, produced by a combination of 450 nm blue and 670 nm purple LEDs at a 1 : 3 ratio) at 24 °C and 8 h dark at 20 °C, 40% relative humidity, in a mixture of 50% DSM WNR1 R73454 substrate (Kekkilä Professional, Vantaa, Finland), 25% grade 3 vermiculite and 25% 3–6 mm perlite. Pest management was performed by the introduction of *Stratiolaelaps scimitus* and *Steinernema feltiae* (Koppert Co., Málaga, Spain).

Plants were inoculated at three different developmental stages referred to as prebolting (juvenile plants), bolting (plants on a transition phase between growth stages), and postbolting (mature flowering plants). Under long-day photoperiod conditions, these stages are reached at 18, 25 and 32 days after-sowing (a.s.), respectively. As bolting can be used as indicator of the vegetative to reproductive phase transition [26], each stage could be associated with different plant stages: vegetative growth, phase transition, and reproductive growth. Following the growth stages described by Boyes *et al*. [29], prebolting corresponded to stage 1.06, bolting to stage 5.10, and postbolting to stage 6.00.

### Inoculation procedure

For all experiments and data analysis, except the hypothesis-validation experiments (i.e. the SA treatment experiment and the viral spread experiment), we used the TuMV lineages generated in a previous study [15]. These viruses were evolved in *A. thaliana* hosts at the three aforementioned developmental stages. During the evolution experiment, 3 lineages were established for each host developmental stage and 10 plants were inoculated per lineage. For more details on the evolution experiment, see Melero *et al*. [15]. The original TuMV isolates used for the evolution experiment were s follows. (i) TuMV-AS, which came from strain YC5 (GenBank, AF53055.2) originally obtained from calla lily (*Zantedeschia* sp.) and cloned under the 35S promoter and *nos* terminator, resulting in the p35STunos infectious clone whose transcription product was inoculated into *Nicotiana benthamiana* plants. To produce the TuMV-AS stock, tissue of symptomatic plants was collected, frozen using liquid N_2_, and homogenized with a Mixer Mill MM400 (Retsch GmbH, Haan, Germany) [27]. (ii) TuMV-DV was obtained from the evolution of the TuMV-AS isolate after 12 passages in 5-week-old (prebolting) short-day-grown *A. thaliana* Col-0 plants [28]. In comparison with the TuMV-AS isolate, TuMV-DV had fixed mutations in amino acids T1293I (cylindrical inclusion protein; CI) and N2039H (viral genome-linked protein; VPg) [30]. For the SA treatment experiment we used the original stocks of TuMV-AS and TuMV-DV as inocula. For the viral spread experiment, we only used the original stock of TuMV-AS as inoculum.

Inoculations were performed using homogenized virus-infected tissue preserved at −80 °C. The virus inoculum consisted of 100 mg of homogeneous N_2_-frozen infected tissue mixed with 1 ml of phosphate buffer and 10% carborundum (100 mg ml^−1^). Plants were inoculated mechanically by rubbing 5 µl of the inoculum into three random rosette leaves of the plant. On the viral spread experiment, only one rosette leaf per plant was inoculated.

### Hormone quantification

Aerial tissue was collected and pooled from plants either mock-inoculated or infected at evolution passage five: one pool per each viral lineage (and three pools of mock-inoculated plants for each developmental stage). Hormone extraction and analysis were carried out as described by Durgbanshi *et al*. [31] with few modifications. Briefly, plant tissue was extracted in ultrapure water in a MillMix20 (Domel, Železniki, Slovenia) after spiking with 10 ng of indole-3-acetic acid ([^2^H_2_]-IAA) and 50 ng of the following compounds: abscisic acid ([^2^H_6_]-ABA), salicylic acid ([^13^C]-SA), phaseic acid ([^2^H_3_]-PA), and dihydrojasmonic acid. Following centrifugation, supernatants were recovered, and the pH was adjusted to 3.0. The water extract was partitioned against diethyl ether and the organic layer recovered and evaporated under vacuum. The residue was resuspended in a 10 : 90 methanol : water solution by gentle sonication. After filtering, the resulting solution was directly injected into an ACQUITY SDS ultra-performance LC system (Waters, Riga, Latvia). Chromatographic separations were carried out on a reversed-phase C18 column (50×2.1 mm, 1.8 µm particle size; Macherey-Nagel GmbH, Dueren, Germany) using a methanol : water (both supplemented with 0.1% acetic acid) gradient. Hormones were quantified using a TQS triple-quadrupole mass spectrometer (Waters) with two technical replicates performed for each sample. Multivariate analysis was perform using the factoextra package version 1.0.7 [32] in R version 4.2.0 [33] in RStudio version 2022.7.1.554.

### High-throughput sequencing and differential expression analysis

RNA was extracted from mock-inoculated and evolution passage five (aerial) infected plant tissue using the NZY Total RNA Isolation kit (NZYTech, Lisbon, Portugal). The quality of the RNAs used to prepare RNA-seq libraries was checked with the Qubit RNA BR Assay kit (Thermo Fisher, Waltham, MA, USA). SMART libraries, Illumina sequencing (paired end, 150 bp), and quality checking of the mRNA-seq libraries were performed by Novogene (UK) Co. Ltd. Raw Illumina RNA-Seq data generated for this study are available in the NCBI SRA under BioProject accession PRJNA1150077. Seventeen bases from the 5′ end and 12 from the 3′ of the reads were trimmed with cutadapt version 2.10 [34]. Trimmed sequences were mapped with HiSat2, version 2.1.0 [35], to the ENSEMBL release 47 of the Arabidopsis TAIR10 genome assembly. Read counting in features was performed with htseq-count, using the Arabidopsis Reference Transcript Dataset (AtRTD2) [36] as input annotation file. Differential expression analysis and characterization of differentially expressed genes were performed with DESeq2, version 1.24.0 [37], only considering genes having a total of at least 10 reads for each pairwise comparison. For all results, we only selected DEGs of *P*≤0.05 and ∣log_2_-fold change∣ > 1.5. Functional profiling was performed using the clusterProfiler package version 4.4.4 [38] with R version 4.2.0 in RStudio version 2022.7.1.554.

### TuMV spread experiment

Thirty-six *A. thaliana* plants were inoculated for each time point established at each of the three developmental stages studied: prebolting, bolting, and postbolting. The inoculated leaf was cut off at either 1, 2 or 3 days post-inoculation (p.i.). As positive controls, 24 plants per developmental stage were inoculated without removing the inoculated leaf afterwards. As negative controls, 12 plants for each time point were mock-inoculated, and the inoculated leaf was removed at the same times that the virus-inoculated plants’ leaves were cut. Plants were inspected for signs of infection at 14 days p.i.

### Supplementation with exogenous SA

SA was dissolved in autoclaved Milli-Q water and diluted to achieve concentrations of 0.5 and 1 mM. Plants at the postbolting stage were inoculated with TuMV-AS and sprayed with (i) 0.5 mM SA, (ii) 1 mM SA, or (iii) water (control plants). Plants were sprayed until they were soaking wet, and substrate was humid. In the experiment performed to characterize infection, the exogenous SA was sprayed once per day from the inoculation day (hours prior to inoculation) until the day prior to their collection (at 14 days p.i.). In the experiment measuring offspring production, treatments were applied daily for 14 days p.i. After this period, treatments were applied once every 3 days for an additional 14 days.

### Infection characterization of SA-treated plants

Upon inoculation, plants were daily inspected for visual symptoms for 14 days p.i. and phenotyped following a discrete scale of symptom severity, ranging from absence of symptoms (0) to full necrosis of the plant (5) (see Fig. 1 in Butković *et al*. [39]). The infectivity and severity of symptoms data along 14 days p.i. were used to calculate the area under the disease progress stairs (AUDPS; [40]) and intensity progression stairs (AUSIPS [41]) values, respectively, as described in Butković *et al*. [42]. AUDPS and AUSIPS values were computed using the agricolae R package version 1.3–2 [43] with R version 4.2.0 in RStudio version 2022.7.1.554.

**Fig. 1.**
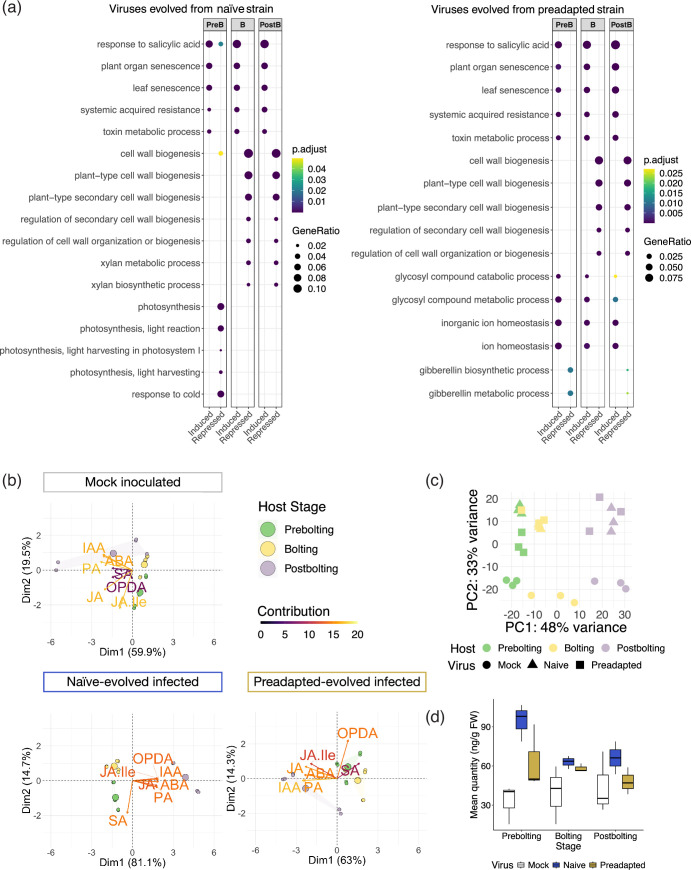
Transcriptional and hormonal response of *A. thaliana* TuMV infection depending on the host developmental stage. (**a**) Gene ontology analysis of differentially expressed genes (DEGs). DEGs upon infection with the naïve-evolved lineages (left plot) and for the preadapted-evolved lineages (right plot). Each column represents the DEGs at each developmental stage. PreB, prebolting hosts; B, bolting hosts; PostB, postbolting hosts. Induced and repressed genes are shown for each developmental stage. The size of the dots is proportional to the ratio of genes for each biological category, and the colour represents the *P*-adjusted values. (**b**) Principal component analysis (PCA) of hormone profiles. Plots are separated for each virus status: mock-inoculated plants, naïve-evolved infected plants, and preadapted-evolved infected plants. Dots represent individuals (composed of pools of plants): prebolting plants in green, bolting in yellow, and postbolting in purple. Arrows represent the variables analysed. The colour of the arrow represents the contribution of each variable to the component on the PCA. (**c**) PCA of the transcriptional response. Samples are differentiated by (**i**) host developmental stage at the time of infection (prebolting in green, bolting in yellow, and postbolting in purple) and (ii) inoculum used (circles for mock-inoculated plants, triangles for plants infected with the naïve-derived viral lineages and squares for plants infected with the preadapted-derived lineages). (**d**) Levels of the hormone SA in mock-inoculated plants (in white), plants infected with naïve-evolved viruses (in blue), and plants infected with preadapted-evolved viruses (in ochre).

### Seed collection

Ten plants were inoculated at each developmental stage for each evolved viral lineage. At 14 days p.i., the virus-inoculated non-symptomatic (considered as non-infected) plants were discarded, and the symptomatic infected ones were maintained in growing chambers until seed production. Total seeds produced were then collected for each individual plant separately, and later counted or weighed to obtain an estimation of the number of seeds produced by each plant considering that, on average, a single seed weighs ~20 µg [44,45]. Mock-inoculated plants were used as a control.

For the plants treated with exogenous SA, 32 plants were mock-inoculated and sprayed with water, 40 were mock-inoculated and sprayed with SA 1 mM, 36 were inoculated with TuMV-AS and sprayed with water, and 36 were inoculated with TuMV-AS and sprayed with SA 1 mM. The same procedures as previously described were used for seed collection.

### Germination test

Three blocks of germination tests were performed. Seeds from random plants inoculated at the same developmental stage were mixed into a pool, without considering the virus isolate used or the stage wherein the virus was previously evolved in. Between 60 and 105 seeds per condition were tested on each germination block. Seeds were sowed on the same substrate and in the same growing chamber conditions used for plant growing. Viable offspring were counted 10 days a.s. Wild-type Col-0 seeds were used as control to measure germination success.

For the seeds coming from the exogenous SA application treatment, seeds from five random plants were selected from each condition (mock water-sprayed plants; mock SA-treated plants, virus-inoculated water-sprayed plants, and virus-inoculated SA-treated plants). A number between 20 and 50 seeds per plant was evaluated. Seeds were sterilized and sowed on plates containing 2.2 g l^−1^ of Murashige–Skoog media with agar, 0.5 g l^−1^ of ethanesulfonic acid, and 10 g l^−1^ of sucrose and maintained in the dark at 25 °C. Viable offspring was counted 10 days a.s. Wild-type Col-0 seeds were used as control to measure germination success.

### Statistical analysis

Differences in viral spread rate were tested by fitting the number of infected plants, *I*, to a log-linear factorial model with a logit-link function by means of a generalized linear model (GLM). In this case, the model equation reads as logit(*I_ijk_*) ∼ *ι+D_i_+ C_j_* + (*D*×*C*)*_ij_ + ε_ijk_*, where *ι* is the grand mean, *D* is the developmental stage, *C* is the time at which the inoculated leaf was removed, both factors considered as orthogonal, and *ε_ijk_* represents the binomial error.

The number of produced seeds (*S*) from plants infected with virus derived from the naïve or the preadapted isolate was fitted to a negative binomial regression with a log-link function using the GLM. The host developmental stage where the virus was tested (*T*), the host developmental stage where the viral lineage was evolved (*E*), and the ancestral viral isolate (*V*) were treated as orthogonal random factors. The full model equation reads as *S_ijkl_* ~ *σ+T_i_+ _Ej_ + V_k_*+(*T*×*E*)*_ij_* + (*T*×*V*)*_ik_* + (*E*×*V*)*_jk_* + (*T*×*E*×*V*)*_ijk_ +ε_ijkl_*, where *σ* corresponds to the grand mean and *ε_ijkl_* represents the negative binomial error.

To test the impact of SA treatment on disease phenotypes, the AUDPS and AUSIPS were calculated from the data for a population of 32 inoculated plants per virus isolate for the water treatment and 36 inoculated plants per virus isolate for each 1 of the 2 SA treatments. A bootstrapping method described by Butković *et al.* [42] was used to estimate 95% confidence intervals. For hypothesis testing, approximated *P* values in pairwise comparisons were obtained by calculating the proportion of overlap of the 95% confidence intervals. *P* values were further adjusted using the sequential Bonferroni’s correction to account for multiple comparisons.

Finally, the number of viable progeny (*R*), was fitted to a GLM with a gamma distribution and a log-link function in which the treatment (*M*) and the infection status (*F*) were treated as orthogonal random factors. The full model equation reads as *R_ijk_* ~ *ρ+M_i_+ F_j_* + (*M*×*F*)*_ij_ +ε_ijk_*, where *ρ* corresponds to the grand mean and ε*_ijk_* represents gamma-distributed errors.

In all analyses, post-hoc pairwise comparisons were made using the sequential Bonferroni method. Computations were performed using SPSS version 28.0.1.0 (IBM, Armonk, NY, USA) or R version 3.6.1 within RStudio version 1.3.1093.

## Results

### Transcriptional and hormonal responses to viral infection depend on the host’s developmental stage

To understand how hosts respond to viral infection at different developmental stages, we used two approaches. First, we examined the transcriptional response in prebolting, bolting, and postbolting host plants. These plants were inoculated with the 18 viral lineages aforementioned. A gene ontology (Fig. 1a) analysis of induced and repressed DEGs indicated that the responses to SA were affected; the SA responses were induced at all three developmental stages for the infections with the naïve-derived lineages. We also found that functions related to cell wall biogenesis were repressed in response to infection. This downregulation was observed for all the lineages in both bolting and postbolting hosts but was absent in prebolting hosts. Bolting and postbolting plants shared more similar profiles in terms of the most overrepresented biological categories for the DEGs identified compared to prebolting hosts, which showed distinct functional profiles. However, a principal component analysis (PCA) for the whole transcriptional response showed that prebolting and bolting plants induced a more similar general response (Fig. 1c). Infection status can be distinguished in the PCA for the transcriptional response, as there is a clear grouping of mock-inoculated plants versus infected ones. A clustering of the samples depending on the developmental stage in which they were inoculated was also observed. In particular, postbolting hosts appeared to be more distant from the other two developmental stages than bolting and prebolting juvenile hosts were from each other.

Second, we studied the hormonal response to infection. Phytohormones orchestrate plant development and responses to both biotic and abiotic stressors [46]. We measured a comprehensive set of hormones of plants differing in (i) their infection status (mock versus infected) and (ii) the developmental stage at which they were inoculated. We performed a PCA on the data to generate a hormone profile (Fig. 1b). Similar to the PCA of the transcriptional response, the hormonal responses of prebolting and bolting hosts were more alike compared to those of postbolting hosts. Infection led to distinct changes in hormone patterns, notably in SA, which showed a pattern change in plants infected with both viruses. Plants infected with viral lineages evolved from a preadapted ancestor also exhibited altered oxo-phytodienoic acid (OPDA) profiles.

### TuMV spreads faster in mature plants

**Fig. 2.**
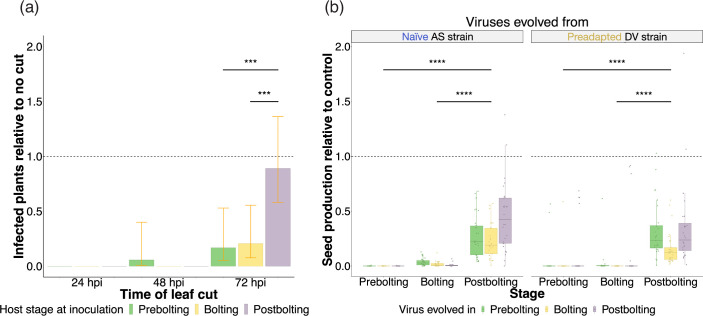
Impact of host developmental stage on virus movement and offspring production. (**a**) Proportion of infected plants after removing the inoculated leaf at different time points (abscissa) in comparison with plants for which the leaf was not removed. Colours represent the host developmental-stage at which the experiment was done (i.e. at the time of inoculation): prebolting plants in green, bolting plants in yellow, and postbolting plants in purple. Error bars represent ±95% CI. (**b**) Box plot representation of the number of seeds produced by each infected plant relative to seed production of mock inoculated plants on each developmental stage. Boxes covers the interquartile range and the horizontal lines correspond to the median. On the abscissa, plants are separated by the developmental stage at which they were infected. For each developmental stage, data are grouped depending on the evolved viral inoculum used for the experiment. In green, there are plants infected with lineages evolved on a prebolting stage, in yellow the ones infected with lineages evolved on a bolting stage, and on purple the ones infected with viral lineages evolved on a postbolting stage. Dots represent individual values of relative seed production for each individual plant. In all cases, asterisks indicate the significance between host developmental stage when inoculated: ****P<0.0001; ***P< 0.001; **P<0.01; *0.01<*P*<0.05.

As we observed a transcriptional downregulation of cell wall biosynthetic genes in bolting and postbolting plants, we sought to investigate whether the higher viral susceptibility of mature plants could be attributed to faster viral spreading between cells and through the whole plant. We hypothesized that a compromised cell wall might facilitate the virus’ exit from the initially inoculated leaves and the subsequent establishment of a systemic infection. To test this hypothesis, we analysed the time required for the virus to spread within the host by inoculating leaves with a naïve isolate of TuMV at the three different host developmental stages -prebolting, bolting, and postbolting- and removing the inoculated leaf at various time points p.i. [47,48]. When the inoculated leaves were removed 1 or 2 days p.i., we observed that almost no plant at any developmental stage showed signs of infection. However, when we deferred the removal of the inoculated leaves to 3 days p.i., there was a noticeable lower incidence of infection in plants at the prebolting (mean frequency ±SE: 0.08±0.05) and bolting stages (0.11±0.05). The number of infected postbolting plants (0.56±0.08) was significantly higher compared to both previous stages (*P*<0.001 in both cases) (Fig. 2a). This higher rate of infection observed in postbolting plants was slightly lower, yet not significantly different, than observed in postbolting plants that had their inoculated leaves intact (0.6±0.1, *P*=1.000), thereby corroborating the enhanced mobility of TuMV in hosts at this stage. Our results thereby point out that, in mature plants, TuMV exits faster from the inoculated leaf and establishes a systemic infection more expeditiously.

### TuMV infection is less virulent in mature plants

The plant’s cell wall plays a vital role in seed development. During reproduction, the reshaping of the cell wall becomes crucial for the expansion and steering of the pollen tube, fertilization of the ovule, and the formation of the seed coat [49]. Mutations that interfere with the biosynthesis or alteration of the cell wall have the potential to hinder these processes and subsequently lower the yield of seeds [50]. Hence, we wondered if TuMV virulence, defined as the reduction in the host offspring caused by infection, was affected by the host developmental stage at which plants are infected. Recall that TuMV is a sterilizing virus [24,25].

We observed clear differences in seed production depending on the developmental stage at which plants were infected (Fig. 2b). In particular, a very reduced proportion of plants infected at prebolting juvenile stage produced seeds (9.09%). By contrast, approximately half of the plants infected at a bolting stage produced seeds (56.80%) and almost all infected plants at a postbolting stage produced seeds (99.42%). Fertility rate (i.e. the proportion of plants producing offspring) values for specific virus lineages and host developmental stages can be seen in Table 1. For all developmental stages, infected plants produced a smaller number of seeds than their relative mock-inoculated plants. Within the infected plants, there was a significant effect of the host developmental stage on the production of seeds: it was higher in postbolting infected plants than in bolting and prebolting hosts, both for viral lineages evolved from the naïve (*P*<0.001) and the preadapted (*P*<0.001) TuMV isolates (Fig. 2b). The developmental stage that viral lineages were evolved in also had an effect on the virus virulence. For lineages derived from the naïve isolate, postbolting hosts infected with postbolting-evolved lineages had a significantly higher production of seeds than when infected with bolting-evolved lineages (*P*<0.001) or prebolting-evolved ones (*P*<0.001).

**Table 1.** Fertility rates calculated for each TuMV isolate inoculated on each host developmental stage. Ninety plants were inoculated for each condition. The total number of infected symptomatic plants is shown and, out of those, the number of plants producing seeds*

Isolate	Stage	Total infected	Infected fertile	Fertility rate†
Naïve	Prebolting	63	5	0.09±0.07
	Bolting	84	70	0.83±0.08
	Postbolting	87	86	0.98±0.04
Preadapted	Prebolting	73	7	0.11±0.07
	Bolting	86	29	0.3±0.1
	Postbolting	86	86	0.99±0.02

*Mock-inoculated plants at each developmental stage showed no symptoms of infection and were all fertile (fertility rate 0.99±0.02).

†Fertility rates are calculated using the LaPlace estimator for a frequency, errors represent the adjusted Wald 95% CI.

Finally, we tested the seeds’ viability (i.e. the proportion of seed that germinated) (Table 2). Seeds produced by postbolting plants were more viable: germination of seeds was higher in postbolting (mean±sd; 0.8±0.2) than in bolting (0.57±0.05) and prebolting plants (0.30±0.01). Hence, the fitness of infected plants is higher if they are infected at later developmental stages; the virulence is reduced in mature plants.

**Table 2.** Germination rates of seeds coming from infected plants at the three developmental stages. Three independent germination tests were performed. Data from the first test on prebolting plants were discarded because asymptomatic plants were noninfected. Germination rate values were normalized to the germination rates of noninfected plants

Stage	Test	Sowed	Germinated	Germination rate*	Relative rate
Prebolting	2	60	16	0.3±0.1	0.327
	3	82	20	0.25±0.09	0.298
Bolting	1	60	27	0.5±0.1	0.530
	2	100	46	0.5±0.1	0.540
	3	105	58	0.55±0.09	0.646
Postbolting	1	60	50	0.8±0.1	0.936
	2	100	52	0.5±0.1	0.592
	3	105	76	0.76±0.08	0.859
Control	1	60	51	0.84±0.09	
	2	100	86	0.85±0.07	
	3	105	93	0.88±0.06	

* Fertility rates are calculated using the LaPlace estimator for a frequency, errors represent the adjusted Wald 95% CI.

### Exogenous application of SA increases postbolting plants’ defences but decreases their fitness

**Fig. 3.**
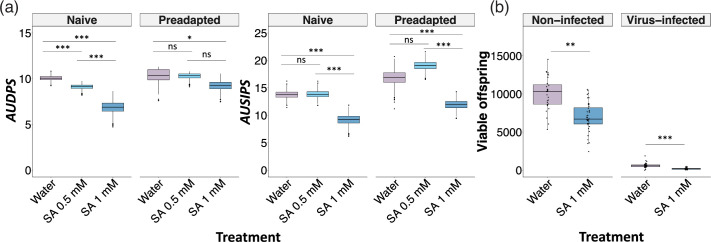
Impact of exogenous application of salicylic acid on susceptibility to infection and production of viable progeny. (a) Box plot representation of bootstrapped data for the progression of the infection (AUDPS, left panel) or of the symptoms (AUSIPS, right panel) of postbolting plants supplemented with water (in purple) or two different concentrations of SA (0.5 mM in light blue and 1 mM in dark blue). Boxes represent the interquartile range, with the horizontal line corresponding to the median values. Data are divided for plants inoculated with lineages derived from the naïve or the preadapted viral isolates. For the naïve-derived lineages, the AUDPS 95% CI were: for water 10.042–10.070; for SA 0.5 mM 9.095–9.131; and for SA 1 mM 6.824–6.905. For the preadapted-derived lineages, they were: for water 10.325–10.403; for SA 0.5 mM 10.262–10.301; and for SA 1 mM 9.202–9.267. In the AUSIPS dataset, the 95% CI for the naïve-derived lineages were: for water 13.716–13.806; for SA 0.5 mM 13.815–13.900; and for SA 1 mM 9.136–9.254. For the preadapted-derived lineages, they were: for water 16.741–16.903; for SA 0.5 mM 18.992–19.098; and for SA 1 mM 11.892–11.994. (b) Number of produced seeds that were viable in non-infected or TuMV naïve-infected plants supplemented with water or 1 mM of SA. In all cases, asterisks indicate the significance between host developmental stage when inoculated: ***P<0.001; **P<0.01; *0.01<*P*<0.05.

Our study of the transcriptional and hormonal responses suggested an involvement of SA in the stage-dependent susceptibility to TuMV infection. Note that the induction levels of SA differ with the host developmental stage at the time of infection. In particular, the induction of this hormone was lower in mature plants compared to younger ones, for both the evolved naïve and preadapted TuMV isolates (Fig. 1d). SA plays a crucial role in local and systemic resistance against pathogens, as well as in systemic acquired resistance (SAR), a ‘whole-plant’ response that occurs following an earlier localized exposure to a pathogen [51,52]. Hence, we wondered whether by increasing the levels of this hormone, reproductive flowering plants would alter the defence/reproduction balance. To test this, we applied exogenous SA to postbolting plants and inoculated them with both the naïve and preadapted TuMV isolates (Fig. 3a). Disease phenotypes (the progression of infected plants, AUDPS, in the population; or their symptoms, AUSIPS; see Methods) indicated that infections progressed more slowly and were less severe in SA-treated plants in comparison with water-sprayed control plants. The mitigation of viral infection in SA-treated plants was more intense for the higher concentration treatment (1 mM).

We then tested the effect of the SA supplementation on the fitness of postbolting mature plants (Fig. 3b). To do so, we inoculated postbolting plants with the naïve TuMV isolate or with buffer (mock), treated with 1 mM SA or water (control), and collected seeds. We found that SA-treated plants produced fewer seeds than control plants, both in mock- (*P*=0.003) and virus-inoculated plants (*P*<0.001). These results suggest that the increased susceptibility in postbolting flowering plants could be compensated for by a higher production of viable offspring than would be produced if the defence response was stronger.

## Discussion

Our results highlight that hosts at later developmental stages suffer from a significant reduction in the expression of genes related to cell wall biosynthesis and metabolism in response to viral infection. The secondary cell wall can play an important role in disease outcomes [53]. To establish a successful systemic infection, plant viruses need to spread along the entire host, moving from one cell to another through the plasmodesmata and further disseminating to distally located tissues through the phloem [54]. Most plant viruses possess a class of structurally diverse proteins known as movement proteins. These proteins may modulate the size exclusion limit of the plasmodesmata [55] and facilitate the virus’s movement through the plant [56,59]. The cell wall identity is a key factor for virus movement [60,61], as changes in the composition of this barrier can modulate both growth and defence processes [62,63].

TuMV infection downregulates cell wall functions [64]. The transcriptomic analyses of viral adaptation highlight cell wall modification as an overrepresented category upon potyviral infection [65]. We show that cell wall processes are specially repressed in both bolting and flowering plants. We hypothesize that the loosening of cell walls on these hosts may facilitate the deformation of plasmodesmata and enable the faster spread of the virus from one cell to another and, ultimately, to the entire host via the phloem. Some viruses, including TuMV, could potentially move systemically not only through the phloem but also through the xylem [66,67]. This implies that the xylem transport of the virus may also be an important factor for systemic spread of the virus. In this case, downregulation of secondary cell wall-related genes on bolting and postbolting hosts may lead to more permeable secondary cell walls on these hosts and facilitate virus transport through xylem vessels. In line with our results, López-González *et al.* [68] showed that TuMV-infected plants downregulate genes related to the formation of the secondary cell wall. They found that *IRREGULAR XYLEM 9*, a gene related to xylan synthesis (a key component of the secondary cell wall), was strongly downregulated upon TuMV infection. This downregulation was correlated with the reduction of xylan in these infected plants. In our analysis, this gene is among the most downregulated cell wall-related genes in response to all TuMV lineage infections in both bolting and postbolting plants. Further investigation will be needed to unravel the specific mechanisms leading to the scenarios observed in our study.

Phytohormones are fundamental players in the interaction between development and immune plant processes [46,69]. It is known that defence genes are induced upon TuMV infection, some of them related with SA signalling pathways [70]. We observed significant changes in hormone profiles in response to infection across different developmental stages. Interestingly, changes were driven by OPDA and SA. Exogenous application of SA can limit the spread and accumulation of a virus, and thus ameliorate disease symptoms [71]. Furthermore, exogenous application of OPDA and/or SA has been shown to reduce the systemic movement of plant viruses by inducing cell wall fortification [72]. In our work, the exogenous application of SA to postbolting plants decreases their susceptibility to infection as well as their fertility when infected. This result is in line with our observation of postbolting plants showing higher susceptibility and fertility upon infection, meaning that they showed a tolerance response, understood as a minimized effect of infection on plant fitness [73,74]. Tolerance varies depending on the host and virus genotypes interacting [75].

Here we show that the developmental stage of the host at the time of inoculation is also a key component of its tolerance to viruses. The flowering transition is a critical moment for plant survival, especially for plants that reproduce only once in their lifespan, such as Arabidopsis [76]. Once reproductive development is established, there is a preference for resource allocation at the cost of defence [76]. This can be explained by the fact that activating a defence response tends to occur at the cost of reducing growth and reproduction [17,19, 77, 78]. The strength and shape of these trade-offs are predicted to drive evolution towards a lower or higher resistance in younger or older individuals [79]. Our observations align completely with these statements: hosts that were infected at their reproductive growth stage produced more viable offspring than those infected at a phase of vegetative growth (which yielded almost no seeds); however, in exchange, viruses showed greater success in infecting hosts during the reproductive stage compared to the vegetative one. Montes *et al.* [80] pointed out that the level of tolerance of host populations can drive the evolution of viruses towards certain levels of virulence and plant tolerance and resistance. Our results evidence that the developmental stage wherein a virus evolves can also be a driving factor of its virulence: when a naïve isolate evolved at a postbolting stage, the resulting lineages were significantly less virulent than those evolved at other developmental stages. The reduced virulence of postbolting-evolved lineages, as compared to lineages evolved at other stages, was not observed for viruses originating from an isolate preadapted to prebolting *A. thaliana* plants. This suggests that the previous evolutionary history of the virus constrains its virulence evolution.

## Abbreviations

ABA: abscisic acid
ARR: age-related resistance
AUDPS: area under the disease progression steps
AUSIPS: area under the symptoms intensity progression steps
CI: 95% confidence interval
CI: cylindrical inclusion protein
DEG: differentially expressed genes
GLM: generalized linear model
IAA: indole-3-acetic acid
LED: light-emitting diode lamp
OPDA: oxo-phytodienoic acid
P: probability value or significance level
PA: phaseic acid
PAR: photosynthetically active radiation
PCA: principal component analysis
p.i.: post-inoculation
SA: salicylic acid
SD: standard deviation
SE: standard error
TuMV: turnip mosaic virus
VPg: viral genome-linked protein
